# Online search and activities of parents of children with ADHD: a qualitative study

**DOI:** 10.1186/s13034-025-00886-5

**Published:** 2025-03-24

**Authors:** Marie Bringer, Sylvain Bodard, Ana Moscoso, Anne Revah-Levy, Diane Purper-Ouakil, Eric Acquaviva, Richard Delorme, Benjamin Landman, Jordan Sibeoni

**Affiliations:** 1 Université de Paris Cité, Paris, France; 2 Sorbonne Université, CNRS, INSERM, Laboratoire d’Imagerie Biomédicale, Paris, France; 3 AP-HP, Hôpital Necker Enfants Malades, Service d’Imagerie Adulte, Paris, France; 4 Center for Transplantation Sciences, Massachusetts General Hospital, Harvard Medical School, Boston, MA USA; 5 ICAR UMR 5191, CNRS, ENS de Lyon, Université Lyon 2 FR, Lyon, France; 6 Service Universitaire de Psychiatrie de l’Adolescent, Argenteuil Hospital Centre, Argenteuil, France; 7 Médecine Psychologique de l’Enfant et de l’Adolescent, Centre Hospitalo-Universitaire de Montpellier, Montpellier, France; 8 INSERM U 1018 Centre de Recherche en Epidémiologie et Santé des Populations (CESP), Hôpital Paul Brousse, Villejuif, France; 9 ECSTRRA Team, UMR-1153, Université de Paris, Inserm, Paris, France; 10 AP-HP, Hôpital Robert Debré, Service Universitaire de Psychiatrie de l’enfant et l’adolescent, Paris, France

**Keywords:** ADHD, Family, Mental health knowledge, Qualitative study

## Abstract

**Background:**

Parents’ perceptions of their child’s mental health play a crucial role in their decision to seek mental health services. Additionally, mental health literacy, which includes knowledge about mental health disorders, is essential for identifying, managing, and preventing mental health problems. Online health information searches are a vital resource for parents of children with attention deficit hyperactivity disorder (ADHD)– one of the most common neurodevelopmental disorders– as they provide emotional support and information on risk factors, treatments, and prognosis. However, while online resources are widely used, little is known about how parents navigate, interpret, and integrate this information into their care decisions. This study explored parents’ lived experiences of seeking ADHD-related information online, examining how these searches shape their perceptions, decision-making, and interactions with healthcare professionals.

**Method:**

This qualitative study followed the Inductive Process to analyze the Structure of lived Experience (IPSE) approach. Twenty parents of children with ADHD were recruited using a purposive sampling strategy, and data were collected through semi-structured interviews until saturation was reached. Data analysis was conducted using a descriptive and structuring procedure to identify key experiential themes.

**Results:**

Data analysis produced three central experiential axes: [1] Internet and the care pathway; [2] Internet knowledge and its supporting role; and [3] Internet and discordant discourse on ADHD between health professionals. Most parents reported using social networks as a crucial source of support, guidance, and mutual aid. Specifically, online parent groups helped them navigate obstacles in the care pathway, manage conflicting perspectives on ADHD, and alleviate feelings of guilt.

**Conclusion:**

Healthcare professionals and stakeholders should consider the impact of web-based resources on parental decision-making and work towards improving the accessibility and reliability of online health information.

**Supplementary Information:**

The online version contains supplementary material available at 10.1186/s13034-025-00886-5.

## Introduction

Attention deficit hyperactivity disorder (ADHD) is a neurodevelopmental disorder characterized by the presence of developmentally inappropriate levels of hyperactive-impulsive and/or inattentive symptoms for at least 6 months in different settings [1]. These symptoms have a direct negative impact on social, academic, and professional activities [1, 2]. ADHD has a global incidence estimated between 5% and 8% [3] and is associated with a heightened risk of poor developmental outcomes [4, 5].

Parents of a child with ADHD self-reported higher rates of psychopathology symptoms than parents of children without ADHD (d = 0.39, 95% confidence interval [0.31, 0.48], *p* <.001, k = 32) [6]. Parental and familial factors are associated with the diagnosis and outcomes of ADHD through multiple pathways, such as direct effects of adverse parenting practices or shared risk factors [7]. Focusing on parents’ experiences is essential since educational and family modalities influence the affective coping of children with ADHD [7]. Parent-centered interventions have greater effects on behavioural symptom improvement [8] and parents’ perception of their child’s mental health status is a predictor and prerequisite for mental health service use [7–11]. In the context of limited mental health knowledge, many studies have highlighted the role of social support in parents’ decision to seek care [11–13]. Whether before or after the diagnosis and treatment of ADHD, the issue of health literacy– that is, the ability of individuals to obtain, understand, and communicate health-related information necessary to make informed decisions– remains crucial [14, 15]. For parents of children with ADHD, mental health literacy is central, and web resources are one of the main sources of information used by parents to learn about their child’s mental health challenges [16].

The number of websites focusing on health is expanding rapidly, and several are entirely dedicated to parents [17, 18]. Before bringing their child to a medical appointment, more than one in five parents, searched online, either to seek a diagnosis (52.3%) or to obtain information on prevention (21.9%) [19, 20]. Most of them reported the information found online to be useful (57%) and the accessibility of resources about children’s mental health to be good or very good [21]. However, few parents use structured strategies for their online research, and many request the help of health professionals to direct themselves to high-quality websites [22]. Web resources are also used to connect with other people in similar situations to alleviate the uncertainty and anxiety of parents about their children’s mental health [12, 23]. Parents turn to the internet to seek information about ADHD, and in 5.9% of situations, this will be their only source of information [12]. Together, these elements — parental perceptions, mental health literacy, and online health searches — form a complex but interdependent framework that highlights the need for accessible, evidence-based online resources to empower parents, enhance their confidence in managing their child’s condition, and ultimately improve outcomes for children with ADHD [24]. To date, only a few studies have explored the online information-seeking behaviors of parents of children with ADHD [25]. Given the increasing reliance on digital resources for health-related decision-making, online information has become a critical tool for families navigating their child’s diagnosis and care pathway, yet no studies have used a qualitative approach to explore how parents seek, interpret, and use online information on that matter.

This study therefore aimed to fill this gap by providing a contextualized understanding of parents’ lived experiences regarding their online searches and activities related to ADHD. The purpose was to offer new insights into the role of online knowledge in shaping parental decision-making. By exploring parents’ experiences, needs, and challenges in seeking ADHD-related information online, this research could help inform both clinical practice and the development of more accessible, evidence-based digital health resources.

## Methods

This exploratory qualitative study used the Inductive Process to analyze the Structure of lived Experience (IPSE) approach [26]. IPSE uses a descriptive phenomenological approach and relies on a constructivist paradigm and an inductive process to capture individuals’ lived experience and to produce the structure of their experience. Five stages, described below, guide the research process. This study report adheres to the COREQ guidelines [27], and a local ethics committee (Comité d’Evaluation de l’Ethique des Projets de Recherche de Robert Debré) has approved all procedures (CEER-RD). This study was conducted in compliance with the ethical regulations and guidelines applicable in France for this type of research and population [28]. As the participants were parents and not patients, no additional ethical approvals were required beyond the standard framework. Written informed consent was obtained from all participants prior to their involvement in the study. The study occurred in the child and adolescent psychiatric department of a Parisian children’s hospital.

**Stage 1: Setting up a research group**. Our research group included a psychiatric trainee (MB) who conducted the interviews, three child and adolescent psychiatrist (CAP) specialists in ADHD (BL, EA, and AM), two CAPs with expertise in qualitative methods (JS and ARL), and a physician from radiology (SB) to avoid confining the results to the sole perspectives of CAPs.

**Stage 2: Ensuring the originality of the study**. Two group members systematically reviewed the qualitative and quantitative literature to confirm the study’s relevance and originality. They verified that no qualitative study in France about this specific topic had been conducted among parents of children with ADHD. To ensure that the other group members could remain inductive and open to novelty, they had access to this review only after they had completed the data analysis.

**Stage 3: Recruitment and sampling**,** aiming for exemplarity**. All parents included in the study had a child with ADHD diagnosed according to Diagnostic and Statistical Manual of Mental Disorders, 5th Edition criteria. A semi-structured interview using the Schedule for Affective Disorders and Schizophrenia for School-Age Children– Epidemiologic Version [29] was used to confirm the diagnosis. Participating parents had to have previously or be currently still seeking information on the internet about their child’s condition and had to speak French fluently. We used a purposive sampling strategy with maximum variation [30] to recruit parents who differed by sex, age, family status, duration of child’s disease and diagnosis delay, type of treatment, and co-occurring disorders. The CAPs working in the child and adolescent psychiatry department were responsible for identifying and inviting parents to participate in the study. The researchers provided the CAPs with the study design and inclusion criteria, and regular exchanges took place with them throughout the data collection period to ensure sample diversity. After initial recruitment, we specifically asked the CAPs to identify more fathers to balance the overrepresentation of mothers.

Sample size was not defined in advance but estimated through the principles of “information power” [31], based on the criteria, quality of dialogue during the interview, the aim of the study and the sample specificity Data collection and analysis were considered complete when the research group considered that the findings obtained provided a sufficient explanatory framework for the data collected, that is reaching theoretical sufficiency [32].

**Stage 4: Data collection**,** access to experience.** One-on-one interviews were conducted either online or in the child and adolescent psychiatric department at the convenience of the parents and the main investigator of the study (MB). The latter had no contact with the parents before the study. Semi-structured interviews were conducted using an open-ended approach [32], guided by an interview framework built from the analysis of two pilot interviews and insights from preparatory meetings between the researchers and the CAPs. The interview guide included six open-ended questions: *Can you tell me how your first concerns or doubts about your child’s difficulties began? Can you tell me in more detail how the search for information online went? How do you rate the quality of the websites you visited? How do you compare the information you find with the medical knowledge provided? What effect has this research and information had on you? How do you continue to use the Internet for information?* These questions were selected as areas to explore because during the pilot interviews they provided rich and spontaneous narratives related to the aim of our study. However, the interviewer used an interactive conversational style [33] and within the IPSE approach, this interview guide should be seen– and serves only– as a narrative support to help the participants to recount their experience as they wish and see fit. In practice, the interviewer used phenomenological and experiential prompts to explore in each area the lived experience of participants and obtain a rich narrative of experience.

The interviews lasted 35–50 min. They were audio-recorded and transcribed into anonymized transcripts, including the participants’ expressive nuances.

**Stage 5: Data analysis**. The IPSE analytic process is a rigorous procedure that relies on an inductive, phenomenological method. In practice, the analysis had two stages: one stage of independent work by MB, JS, and BL, aided by Nvivo software [34], and the other by all researchers pooling the data collectively. In the individual procedure, MB, JS, and BL independently and simultaneously conducted a systematic descriptive analysis to convey each participant’s experience. This involved for each interview: [1] listening to the recorded interview twice and reading the transcript three times; [2] exploring the experience word by word, that is, cutting up the entire text into descriptive units; [3] regrouping the descriptive units into categories. During the group process, the three researchers met regularly with the rest of the research team, who had familiarized themselves with the data– by listening and reading all the interviews as many times as needed– to discuss and refine the analysis. These two-hour meetings, dedicated to reviewing and structuring the emerging findings, began after the analysis of five interviews. During these meetings, the researchers first conducted the structuring phase, that is, to regroup the categories into axes of experience, constructed such that each could be linked to its subjacent categories, and to determine the structure of lived experience characterized by the central axes. They then conducted the practical phase, the process of triangulating the data with what is known in the comprehensive literature on the topic, making it possible to identify the novel contributions and unique findings of the results.

### Rigor criteria

To ensure the rigor of our analysis and the trustworthiness of our results, we applied several established qualitative research criteria: triangulation, attention to negative cases, and reflexivity.

The concept of triangulation refers here to triangulating data sources, that is, the use of multiple data sources (parents’ interview and comprehensive literature on the topic ) and investigator triangulation. Three researchers were directly involved with data collection and individual analytical procedures, both being rigorous procedures to ensure a global understanding of the phenomenon under study [35]. Attention to negative cases involves systematically searching for data that challenge emerging results. In this study, particular attention was paid to the cases in which new elements differed radically from the emerging structure of the experience. These negative, sometimes contradictory, cases were integrated into the results.

Reflexivity is the process of researchers critically examining their own influence on the research process, including how their positionality, assumptions, emotions, and interactions with participants shape the findings [36]. In our study, reflexivity was a continuous effort, individually through personal notes and within the group through open discussions during regular meetings in which researchers reflected on their role and the potential impact of their perspectives on the results.

These practices were systematically integrated into our group process, fostering transparency and ensuring that our findings were as robust and nuanced as possible.

## Results

Twenty-three parents were approached and 20 parents (1 couple, 14 mothers, 4 fathers) were included in the study (Table 1). Among the first 15 parents interviewed, only one father was interviewed along with his wife. Four parents were initially interested in participating but then did not go through without giving any specific reason other than lack of availability.

**Table 1:** Characteristics of participating families and fathers

Parents	Sex of the child concerned			Diagnosis		Nb of children parental couple	Rank of siblings of child concerned			Member of the couple interviewed	Profession of the parent interviewed			Couple marital status	
M 1	B			ADHD + ODD		1	1			Mother	Human resources consultant			Separated or divorced	
M 2	B			ADHD + ODD + Specific learning disorders		3	-			Mother	National Education			Couple	
MF 3	B			ADHD		2	1			Couple	Father: computer scientist/Mother: housewife			Couple	
M 4	B			ADHD		2	2			Mother	Actress			Separated or divorced	
M 5	B			ADHD		3	2			Mother	Notary			Couple	
M 6	G			ADHD		4	2			Mother	Childcare assistant			Separated or divorced	
M 7	G			ADHD		2	1			Mother	Nurse			Couple	
M 8	G			ADHD + ASD		2	2			Mother	Childcare assistant			Couple	
M 9	G			ADHD		2	1			Mother	Communication			Couple	
M 10	G			ADHD		2	1			Mother	Preschool Teaching Assistant			Couple	
M 11	B			ADHD		2	2			Mother	Pharmaceutical assistant			Couple	
M 12	B			ADHD + Developmental Coordination Disorder		2	1			Mother	Childminder			Couple	
M 13	B			ADHD		2	2			Mother	Accountant			Couple	
M 14	B			ADHD		2	1			Mother	Management assistant			Separated or divorced	
M 15	B			ADHD + Specific learning disorders		2	1			Mother	Social worker			Couple	
F 16	G			ADHD		4	2			Father	Paramedic			Separated or divorced	
F 17	B			ADHD + Developmental Coordination		2	1			Father	Computer scientist			Couple	
F 18	B			ADHD		2	2			Father	Retired			Couple	
F 19	B			ADHD		2	2			Father	Actor			Separated or divorced	

ADHD: Attention deficit hyperactivity disorder; ODD: oppositional defiant disorder; ASD: autism spectrum disorder; G: girl; B: boy

Data analysis produced a common structure of lived experience based on three axes of experience of [1] Internet and the care pathway; [2] Internet knowledge and its supporting role; and [3] Internet and discordant discourse on ADHD between health professionals. Transcript excerpts presented below have been selected to exemplify the themes described and translated into English for the sole purpose of this article.

Other relevant quotations from the interview transcripts, translated from French to English specifically for this article, are presented in supplementary material (Table S1).

### Internet and the care pathway

#### The first steps: symptoms as keywords and websites

Parents described looking online for symptoms similar to those exhibited by their child as one of the first steps in online research. They all mentioned an experience of floundering by entering *symptom keywords* in the search bar, the most common being “child’s anger”, “hyperactive child”, “hyperactivity”, “child’s lack of concentration” and “young child’s agitation”. It was through the lens of these keywords that they then reached and consulted websites related to ADHD, their first informal confrontation with the diagnosis.

I did my research when I had doubts, in fact, I immediately went on the Internet to see the exact symptoms etc. and well, I recognized my son. (M11)

Most of the parents shared the experience of consulting the same websites:

- Websites of patient and parent organizations, such as “Hyper Supers TDAH France”, allow identification of the symptoms described and a first overview of the care pathway.

I immediately joined the ADHD France group on Facebook, and right away, you understand better, (…) We are informed, helped, guided, that’s it. In this group, more than on Google, we are guided well, well-informed, we know what to do and where to go. (M2)

- Official websites, such as the one of the Regional Health Agency that parents came across quite early during their searches, were described as enlightening and informative.
- Other unofficial online resources, most of which they no longer remember.

#### Recognizing the diagnosis without guidance on treatment

Identifying symptoms online was an important first step for parents. They felt encouraged to continue their research they felt encouraged to continue their research due to their interactions with other parents who were experiencing similar situations with their child.

Many parents considered that their online search significantly contributed to a medical validation of their child’s difficulties, making it possible to transform external perspectives into a form of support (recognition, care, school accommodations).

That was the turning point, in fact the diagnosis, that’s the turning point. That means you’re in the right hands, people who know what you’re going through and who will really be able to help you. (M4)

However, parents experienced a lack of signposted care routes online. They also conveyed frustration and distress regarding the absence of clear guidance on diagnostic and care pathways, relying on those around them or those responsible for their child (teachers, speech therapists, pediatricians) to access this information.

The fathers interviewed witnessed the mothers’ struggles with this matter online but did not actively share the same experience.

I let the mother manage, frankly the mother managed a lot, which she blamed me a little for not having participated enough. (F18)

### Internet, knowledge and the supporting role

#### Share and help each other

All the mothers spoke about the major supporting role they found online through social networks and other online fora. They described that when they could not find answers to their questions, social media offered real help and guidance. Some used social networks or fora to learn about ADHD.

The mothers experienced guidance and clarification of the information previously collected through social media. They felt they could escape from wandering, uncertainty, and doubts thanks to these virtual support networks between parents.

Then I go on fora, I listen, I read what other parents are saying, and I compare it to my son’s condition. So, the fora, it’s real-life experiences, you know it’s people who have been through it. I spent hours, really, hours reading all of that, and what helped me the most were the fora. (M11)

Parents notably used Facebook groups for parents of children with ADHD such as “TDAH France HyperSupers”, “ADHD TypiK’AtypiK”. They shared their information and experiences through questions and answers to the community. These search strategies were seen as “more horizontal”,giving parents precise and almost immediate answers to their questions.

Mothers also explained that social networks brought together testimonies that compare experiences that can reassure and guide. They felt relieved to be finally heard and understood by other parents and highlighted the practical information and daily parental guidance via advice from other parents on these networks.

But it’s true that when moms share their daily experiences and give their little tips, like game ideas or using certain resources, you think, ‘Okay, I’ll try this kind of thing, and maybe it’ll work, or maybe not, we’ll see.’ So, these little tips make you think, ‘Well, she has a similar daily life to mine, so maybe it’ll work for us too. (M10)

#### Seeking practical and official ADHD information online

On this matter, again, fathers shared different perspectives. They expressed a preference for official sources, such as medical websites, and reported being cautious about the emotional intensity of parent testimonies or even avoiding parent discussiongroups.

The first research you do is to understand the overall ADHD system, to know what it is exactly, what is happening in your child’s brain (…) And then you are also confronted at certain times with testimonies which can increase your anxiety by saying to yourself we are never going to get out of this, but you have seen this thing it is even worse. But for me to read even worse things doesn’t reassure me, it really worries me (…) For me the main danger of the Internet is people’s judgments and the torrent of emotions and anguish when the sites are only receptacles of anguish, distress, and despair. I can’t do it, it’s too violent, it’s too difficult. (F19)

This axis revealed notable differences and similarities in how fathers and mothers experienced and navigated their child’s ADHD diagnosis and care.

- *Emotional impact and role in the care pathway*: Mothers, driven by guilt, loneliness, and external judgment, took an active role in their child’s care, while fathers remained more distant, often relying on their partners, which sometimes led to tension.
- *Use of online resources*: Mothers predominantly used social networks and fora to seek emotional support and practical advice from other parents in similar situations while fathers preferred official and scientific sources for accurate and factual information about ADHD.
- *Perception of professional guidance*: Mothers aligned their expectations with the advice and guidance found online, sometimes leading to frustration when healthcare professionals failed to meet these expectations. Fathers adopted a more critical stance, focusing on the gaps between professional opinions and the information found online.
- *Coping strategies*: Mothers often used online resources as a way to cope emotionally with the challenges of parenting a child with ADHD, finding reassurance and validation in shared experiences. Fathers, while less involved in emotional exchanges, focused on understanding the condition scientifically to support their children practically.

### Internet and discordant discourse on ADHD between health professionals

#### Contradictory discourses on ADHD

Several parents reported encountering a significant gap between the information available online and the explanations provided by healthcare professionals. Many experienced frustration when their concerns, reinforced by their online searches, were dismissed by professionals.

And here we are confronted with a double discourse. That is to say the discourse of neuropsychiatrists and the discourse of psychotherapists with a psychoanalytic tendency. And who therefore absolutely do not have the same vision of ADHD. (M15)

Some parents felt lost, struggling to navigate conflicting messages and choose between different interpretations of ADHD.

But it’s true that there was a bit of wandering, you know… you feel like you’re left on your own, going from one professional to another without really knowing, without receiving any guidance. (M9)

Others fully aligned with the perspectives shared within their online communities, reinforcing a sense of belonging but sometimes leading to tensions with healthcare professionals over what constitutes legitimate knowledge.

At the same time, some parents chose to step back from online communities, feeling the need to regain critical thinking and form their own perspective.

#### Mismatched expectations and uncertainty

Parents recounted the significant contrast between what they expected from professionals– expectations built from their online interactions with social networks– and what really happened.

Parents expressed feeling emotionally drained by repeated consultations that did not yield the same conclusion and solutions they found online.

All this to say that after everything, I felt like it wasn’t leading anywhere, there wasn’t really a diagnosis for my son, so I put things on hold for a few months because I was just tired, really—I was trying to do what I could. (M5)

## Discussion

Our findings highlight how parents’ online searches for information about ADHD are deeply intertwined with their lived experiences of the diagnostic and care pathway. The results reveal not only the pivotal role of digital resources in shaping their understanding and decision-making but also the emotional and practical challenges they face when navigating conflicting information and professional guidance.

A recent literature review about the mental health knowledge of Internet users highlighted that digital interventions (ranging from parental education modules to consultation of popular health information sites) significantly improved parents’ knowledge scores about ADHD [37]. Another study highlights the importance of online searches for parents of children with ADHD. However, our study provides additional insights by showing how these searches influence not only their understanding of the disorder but also their relationship with healthcare professionals and their care pathway. This difference underscores the importance of considering how parents interpret and integrate information into their decisions, which can have a direct impact on the guidance and effectiveness of medical follow-up.

Our study highlights the supporting role of online social networks and discussion fora, with parents particularly emphasizing the abundance of practical information available on social networks compared to more official sites on ADHD. In addition, receiving advice from other parents in a similar situation was more accepted and more impactful. It has been shown that Facebook remains the preferred social network for parents looking for information on their children’s health [38]. Parents in our study clearly remembered the support groups they joined but struggled to recall non-social network websites, highlighting differences in the efficiency and experience of online searches. Social networks attract intense movements of sharing/claiming and belonging/non-belonging, the question of identity and affiliation within a group of parents of ADHD children becomes central [39].

Our results suggest an intertwining of the search of information and the affiliation through these networks with a community of “parent-peers”, since such affiliation comes with a discourse that informs, guides, supports and advises. In other words, these communities establish a clear discourse on ADHD from which it can be difficult for some members to detach themselves. Some parents described what we call a *belonging and identity trap* [40], referring to a situation where a person feels trapped by their group identity or by the expectations and norms associated with belonging to a particular group.

Our findings highlight how online communities serve as crucial spaces for parents of children with ADHD, providing support, guidance, and a sense of belonging. This aligns with previous research showing that digital communities help alleviate parental guilt and reinforce the neurodevelopmental perspective of ADHD, encouraging parents to take a proactive role in their child’s diagnosis and care [38, 39, 41].

In 2019, a French study found that the discourses from online communities of parents with ADHD were articulated around the alleviation of guilt from families, the neurodevelopmental origin of ADHD, and recommendations to establish a diagnosis, thus encouraging parents to play a proactive role in the care of their child [41]. Digital communities thus give worried parents a place of mutual assistance in a context of distrust of current professional practices [41]. Furthermore, our study supports findings that online platforms contribute to polarized discussions on ADHD treatments, particularly psychostimulants. This is particularly problematic in France since there is a lack of consensus regarding how to consider and approach ADHD not only in the lay public but also among French mental health professionals themselves [42]. A recent study in two French regions confirmed the heterogeneity of ADHD-related knowledge among general practitioners, pediatricians, and CAPs, further complicating parental decision-making [43].

Our findings highlight clear gender differences in the use and expectations of online resources among parents of children with ADHD. Fathers in our study tended to perceive social networks as too emotionally charged and subjective, preferring instead the structured, evidence-based content of academic and official websites. On the other hand, mothers predominantly turned to social networks and fora to share experiences, find practical advice, and build a sense of community, which provided emotional reassurance and guidance in navigating the diagnostic and care process. This is consistent with studies indicating that mothers are more engaged in emotional and social aspects of caregiving and often seek peer support when managing their child’s health challenges [38, 39]. Our results therefore suggest distinct patterns that would need to be assessed and investigated in a further quantitative study. These patterns could highlight the importance of tailoring interventions and resources to accommodate the differing needs and roles of both parents. Specifically, targeted support could empower mothers by alleviating emotional burdens and fostering effective coping strategies, while also encouraging fathers to engage more holistically by addressing their preference for evidence-based resources and practical involvement. Understanding these dynamics is crucial for developing family-centered interventions that enhance collaboration between parents and ultimately improve outcomes for children with ADHD.

Online content about ADHD serves as a key reference point for parents as they navigate the different perspectives and medical approaches encountered when consulting specialists. The variety of available information can sometimes lead to confusion, as parents feel compelled to choose between differing explanations and treatment options. This uncertainty is often addressed through exchanges with other parents on social networks, where they seek reassurance, compare experiences, and refine their understanding of ADHD. As highlighted by the study on “Mumsnet” [44], a widely used parenting discussion forum in the United Kingdom, parents actively engage in discussions to validate information, clarify conflicting advice from professionals, and find emotional support. These online interactions help shape their expectations and influence how they interpret and respond to professional guidance [44].

## Strengths and limitations of the study

This is the first French study focusing on the lived experience of online search and activities of parents of ADHD children. The IPSE approach provided a rigorous framework for capturing parents’ lived experiences through iterative analysis and researcher triangulation. However, some limitations must be acknowledged. The sample included only heteronormative and cisgender parents with an overrepresentation of mothers, reflecting real-world caregiving patterns but potentially limiting the diversity of perspectives. Future research should aim for a more diverse and balanced representation. An Australian study looked closely at fathers of children aged 0 to 8 years old and in need of mental health care. This identified various obstacles to fathers seeking help such as the need for control and autonomy in managing problems, a tendency to minimize their struggles, and a feeling of resignation to the idea that nothing will help [45]. This greater passivity of fathers calls into question the mental burden placed on mothers. On a sociological level, this brings a gendered perspective to the interpretation of behaviour. However, mothers and fathers in our study shared an overall common structure of lived experience but with different perspectives, especially about social networks and forum discussions. Indeed, our results suggest key differences between fathers and mothers that we chose to highlight, these results provide relevant insights on how gendered roles and perceptions influence parental engagement and coping mechanisms. However, this qualitative study, with a small sample size and no measurable variables, does not allow for a comparative analysis.

Data collection through semi-structured interviews allowed for in-depth exploration but may have introduced self-selection bias, favoring participants with stronger engagement in online communities. Furthermore, the absence of observational data limits the ability to contextualize online behaviors fully. While the study’s methodological rigor enhances its validity, future research should further diversify participants and adopt mixed-methods approaches to deepen the understanding of parental experiences with online information-seeking.

This qualitative study has some limitations that might hinder its transferability. First, even if online search and activities are now part of daily lives in most countries, it took place in France, and caution is required in transposing our findings to other places because, as discussed above, findings are embedded in the French sociocultural context, in general, and within the French system of child and adolescent psychiatry, in particular. In France, ADHD treatment is embedded in a debate marked by two main theoretical approaches: the neurodevelopmental and psychodynamic currents that not only influence clinical practices but also shape the perception of ADHD in French society. Further research should explore the same research question with parents from other countries.

Second, our specific focus on ADHD raises the question of whether parent’s expectations and patterns of Internet use are comparable across other psychiatric and non-psychiatric conditions. Studying these dynamics in different contexts would help to determine how specific they are to ADHD or if they are part of a broader pattern. Although the number of participants [20] allowed for the collection of rich and diverse data, consistent with the objective of a qualitative study, we acknowledge that the sample size limits the generalizability of the findings to other populations, which should be considered when interpreting findings.

Finally, our study was conducted in a single-center, a university child and adolescent psychiatric department. It would be interesting to conduct a study with a similar design in other settings such as outpatient centers of child and adolescent mental health services and private practices.

## Conclusion

The growing mobilization of families via associative organizations or parent groups contributes to a more active role for parents in help-seeking and managing their children’s conditions. Through their shared experiences and the insights gained from networks, parents further enhance their role as valuable contributors to the information provided to doctors. By highlighting these experiences, this study provides a deeper understanding of how parents navigate online information, reflecting their needs for clear, evidence-based guidance and supportive online spaces.

## Supplementary Information

Supplementary material 1

## Data Availability

No datasets were generated or analysed during the current study.

## Abbreviations

ADHD: Attention deficit hyperactivity disorder
ASD: Autism spectrum disorder
CAPs: Child and adolescent psychiatrists
COREQ: Consolidated criteria for reporting qualitative research
IPSE: Inductive process to analyze the structure of lived experience
ODD: Oppositional defiant disorder

## References

[1] Felt BT, Biermann B, Christner JG, Kochhar P, Harrison RV. Diagnosis and management of ADHD in children. Am Fam Physician 1 Oct. 2014;90(7):456–64. 25369623

[2] Bélanger SA, Andrews D, Gray C, Korczak D. ADHD in children and youth: part 1—Etiology, diagnosis, and comorbidity. Paediatrics Child Health 24 Oct. 2018;23(7):447–53. 10.1093/pch/pxy109 PMC6199644 30681669

[3] WHO. World Health Organization 2019. 2019.

[4] Erlandsson S, Lundin L, Punzi E. A discursive analysis concerning information on ADHD presented to parents by the National Institute of mental health (USA). Int J Qualitative Stud Health Well-being Janv. 2016;11(1):30938. 10.3402/qhw.v11.30938 PMC4823630 27052426

[5] Banaschewski T, Becker K, Döpfner M, Holtmann M, Rösler M, Romanos M. Attention-Deficit/Hyperactivity disorder. Deutsches Aerzteblatt Online [Internet]. 3 mars 2017 [cité 1 déc 2021]; Disponible sur: https://www.aerzteblatt.de/10.3238/arztebl.2017.0149 10.3238/arztebl.2017.0149 PMC5378980 28351467

[6] Faraone SV, Perlis RH, Doyle AE, Smoller JW, Goralnick JJ, Holmgren MA, et al. Molecular genetics of Attention-Deficit/Hyperactivity disorder. Biol Psychiatry Juin. 2005;57(11):1313–23. 10.1016/j.biopsych.2004.11.024 15950004

[7] Claussen AH, Holbrook JR, Hutchins HJ, Robinson LR, Bloomfield J, Meng L et al. All in the family?? A systematic review and Meta-analysis of parenting and family? environment as risk factors for Attention-Deficit/Hyperactivity disorder (ADHD) in children. Prev Sci [Internet] 19 avr 2022 [cité 3 sept 2022]; Disponible sur: https://link.springer.com/10.1007/s11121-022-01358-4 10.1007/s11121-022-01358-4 PMC9017071 35438451

[8] Doffer DPA, Dekkers TJ, Hornstra R, Van Der Oord S, Luman M, Leijten P, et al. Sustained improvements by behavioural parent training for children with attention-deficit/hyperactivity disorder: A meta‐analytic review of longer‐term child and parental outcomes. JCPP Adv Sept. 2023;3(3):e12196. 10.1002/jcv2.12196 PMC10501699 37720584

[9] Haute Autorité de Santé [Internet]. [cité 30 nov 2024]. TDAH de l’enfant et adolescent: former plus de professionnels pour réduire les délais de prise en charge. Disponible sur: https://www.has-sante.fr/jcms/p_3542741/fr/tdah-de-l-enfant-et-adolescent-former-plus-de-professionnels-pour-reduire-les-delais-de-prise-en-charge

[10] Sayal K, Mills J, White K, Merrell C, Tymms P. Predictors of and barriers to service use for children at risk of ADHD: longitudinal study. Eur Child Adolesc Psychiatry Mai. 2015;24(5):545–52. 10.1007/s00787-014-0606-z 25201055

[11] Frauenholtz S, Conrad-Hiebner A, Mendenhall AN. Children’s mental health providers’ perceptions of mental health literacy among parents and caregivers. J Family Social Work Janv. 2015;18(1):40–56.

[12] Yu XH, Zhong L, Huang J. How online ADHD-related information affects Chinese parents’ decisions? World J Pediatr Févr. 2019;15(1):57–65. 10.1007/s12519-018-0207-x PMC6394639 30478599

[13] Abera M, Robbins JM, Tesfaye M. Parents’ perception of child and adolescent mental health problems and their choice of treatment option in Southwest Ethiopia. Child Adolesc Psychiatry Ment Health. 2015;9:40. 26300967 10.1186/s13034-015-0072-5 PMC4546139

[14] Yamashita A, Isumi A, Fujiwara T. Online peer support and Well-being of mothers and children: systematic scoping review. J Epidemiol 5 Févr. 2022;32(2):61–8. 10.2188/jea.JE20200079 PMC8761562 33132282

[15] Cormier E, Park H, Schluck G. eMental health literacy and knowledge of common child mental health disorders among parents of preschoolers. Issues Ment Health Nurs Juin. 2020;41(6):540–51. 10.1080/01612840.2020.1719247 32400237

[16] Mackert M, Mabry-Flynn A, Donovan EE, Champlin S, Pounders K. Health Literacy and Perceptions of Stigma 10 10.1080/10810730.2019.1678705 31630662

[17] Montagni I. Littératie En Santé mentale: de quoi s’agit-il et Pourquoi La Promouvoir? QSP Mai 2023;(46):1–4.

[18] Lambert SD, Loiselle CG. Health Information—Seeking behavior. Qual Health Res 1 Oct. 2007;17(8):1006–19. 10.1177/1049732307305199 17928475

[19] Sbaffi L, Rowley J. Trust and credibility in Web-Based health information: A review and agenda for future research. J Med Internet Res 19 Juin. 2017;19(6):e218. 10.2196/jmir.7579 PMC5495972 28630033

[20] Sebelefsky C, Voitl J, Karner D, Klein F, Voitl P, Böck A. Internet use of parents before attending a general pediatric outpatient clinic: does it change their information level and assessment of acute diseases? BMC Pediatr Déc. 2016;16(1):129. 10.1186/s12887-016-0677-8 PMC4991080 27538782

[21] Montoya A, Hernández S, Massana M, Herreros O, Garcia-Giral M, Cardo E, et al. Evaluating internet information on attention-deficit/hyperactivity disorder (ADHD) treatment: parent and expert perspectives. Educ Health. 2013;26(1):48. 10.4103/1357-6283.112801 23823673

[22] Sim NZ, Kitteringham L, Spitz L, Pierro A, Kiely E, Drake D, et al. Information on the world wide Web—how useful is it for parents? J Pediatr Surg Févr. 2007;42(2):305–12. 10.1016/j.jpedsurg.2006.10.003 17270540

[23] Benedicta B, Caldwell PH, Scott KM. How parents use, search for and appraise online health information on their child’s medical condition: A pilot study. J Paediatr Child Health Févr. 2020;56(2):252–8. 10.1111/jpc.14575 31365171

[24] Sanders MR, Morawska A, Haslam DM, Filus A, Fletcher R. Parenting and family adjustment scales (PAFAS): validation of a brief parent-report measure for use in assessment of parenting skills and family relationships. Child Psychiatry Hum Dev Juin. 2014;45(3):255–72. 10.1007/s10578-013-0397-3 23955254

[25] Sage A, Carpenter D, Sayner R, Thomas K, Mann L, Sulzer S, et al. Online Information-Seeking behaviors of parents of children with ADHD. Clin Pediatr (Phila) Janv. 2018;57(1):52–6. 10.1177/0009922817691821 28183190

[26] Sibeoni J, Verneuil L, Manolios E, Révah-Levy A. A specific method for qualitative medical research: the IPSE (Inductive Process to analyze the Structure of lived Experience) approach. BMC Med Res Methodol. 26 août. 2020;20(1):216. 10.1186/s12874-020-01099-4 PMC7449004 32847514

[27] Booth A, Hannes K, Harden A, Noyes J, Harris J, Tong A. COREQ (Consolidated criteria for reporting qualitative Studies). In: Moher D, Altman DG, Schulz KF, Simera I, Wager E, editors. éditeurs. Guidelines for [internet].porting health [internet].search: A user’s manual [Internet]. 1^[internet].^ éd. Disponible sur: Wiley; 2014. pp. 214–26. [cité 17 août 2024]. 10.1002/9781118715598.ch21.

[28] Légifrance - Publications officielles -. Journal officiel - JORF n° 0056 du 06/03/2012 [Internet]. [cité 19 janv 2025]. Disponible sur: https://www.legifrance.gouv.fr/download/pdf?id=RmLuYk2_t0sMKpcoquw-OSu1fmt64dDetDQxhvJZNMc=

[29] Kaufman J, Birmaher B, Brent D, Rao U, Flynn C, Moreci P, et al. Schedule for affective disorders and schizophrenia for School-Age Children-Present and lifetime version (K-SADS-PL): initial reliability and validity data. J Am Acad Child Adolesc Psychiatry Juill. 1997;36(7):980–8. 10.1097/00004583-199707000-00021 9204677

[30] Patton MQ. The Nature of qualitative inquiry. 3rd edition. 2001.

[31] Morse JM. The significance of saturation. Qual Health. 1995.

[32] Britten N. Qualitative research: qualitative interviews in medical research. BMJ 22 Juill. 1995;311(6999):251–3. 10.1136/bmj.311.6999.251 PMC2550292 7627048

[33] Mason J, May T. Qualitative interviewing: asking, listening and intepreting. Qualitative [internet]esearch in action [Internet]. Sage Publications Ltd; 2002. [cité 6 févr 2025]. Disponible sur:. https://research.manchester.ac.uk/en/publications/qualitative-interviewing-asking-listening-and-intepreting.

[34] Dhakal K. NVivo. Jmla [Internet]. 26 Avr 2022 [cité 21 [internet].v 2024];110(2). Disponible sur: https://jmla.pitt.edu/ojs/jmla/article/view/1271

[35] Carter N, Bryant-Lukosius D, DiCenso A, Blythe J, Neville AJ. The use of triangulation in qualitative research. Oncol Nurs Forum Sept. 2014;41(5):545–7. 10.1188/14.ONF.545-547 25158659

[36] Finlay L. Negotiating the Swamp: the opportunity and challenge of reflexivity in research practice. Qualitative Res 1 Août. 2002;2(2):209–30.

[37] PEYTON D. Do Digital Health Interventions Improve Mental Health Literacy or Help-seeking Among Parents of Children Aged 2–12 Years? A Scoping Review. 2019. 10.3233/SHTI190788 31397317

[38] Frey E, Bonfiglioli C, Brunner M, Frawley J. Parents’ use of social media as a health information source for their children: A scoping review. Acad Pediatr Mai. 2022;22(4):526–39. 10.1016/j.acap.2021.12.006 34906742

[39] Douville L, Rocheleau K, Normand A, L’UTILISATION D’INTERNET ET, DES RÉSEAUX SOCIAUX COMME SOUTIEN AUX PRATIQUES PARENTALES. EXPÉRIENCE DES MÈRES D’ENFANTS D’ÂGE PRÉSCOLAIRE. Revue Québécoise De Psychologie. 2021;42(3):115.

[40] Mounk Y. The identity trap: A story of ideas and power in our time. Random House. 2023.

[41] Dauman N, Haza M, Erlandsson S. Liberating parents from guilt: a grounded theory study of parents’ internet communities for the recognition of ADHD. Int J Qualitative Stud Health Well-being 1 Janv. 2019;14(1):1564520. 10.1080/17482631.2018.1564520 PMC6352942 30696381

[42] Ponnou S. How French media have portrayed ADHD to the lay public and to social workers. 2017; Disponible sur: https://pubmed.ncbi.nlm.nih.gov/28532330/10.1080/17482631.2017.1298244 PMC5510191 28532330

[43] Willig TN, Dajon M, Assathiany R, Brun L, Fourneret P, Massé M, et al. Healthcare pathways and practitioners’ knowledge about ADHD in children. Encephale Août. 2024;50(4):363–72. 10.1016/j.encep.2023.07.005 37718197

[44] Croucher L, Mertan E, Shafran R, Bennett SD. The use of Mumsnet by parents of young people with mental health needs: qualitative investigation. JMIR Ment Health 3 Sept. 2020;7(9):e18271. 10.2196/18271 PMC7499161 32880583

[45] Giallo R, Dunning M, Gent A. Attitudinal barriers to help-seeking and preferences for mental health support among Australian fathers. J Reproductive Infant Psychol 27 Mai. 2017;35(3):236–47. 10.1080/02646838.2017.1298084 29517311

