# Supplementary material for: Online search and activities of parents of children with ADHD: a qualitative study
Source: Child Adolesc Psychiatry Ment Health. 2025 Mar 24;19:31. doi: 10.1186/s13034-025-00886-5 (PMC11934721; doi:10.1186/s13034-025-00886-5)
Supplement: Supplementary file 1 — Supplementary material 1 [file 13034_2025_886_MOESM1_ESM.docx]

**Online search and activities of parents of children with ADHD: a qualitative study**

By Marie Bringer, Sylvain Bodard, Ana Moscoso, Anne Revah-Levy, Diane Purper-Ouakil, Eric Acquaviva, Richard Delorme, Benjamin Landman, Jordan Sibeoni

*1. Supplementary table*

2. *Correspondence to:* Dr Marie Bringer, Service Universitaire de Psychiatrie de l’enfant et l’adolescent, Robert Debré, 48 Bd Sérurier, 75019 Paris, France. Phone number: +33699050771 Email address: [marie_bringer@hotmail.fr](mailto:marie_bringer@hotmail.fr)

**Suppl 1. Table S1: quotations**

1. **Internet and the carepathway**

| *The first steps: symptoms as keywords and websites* | (F18) *“I found it quite easily. It's a shame because I don't remember it at all anymore”* |
| --- | --- |
| *Recognizing the diagnosis without guidance on treatment* | (M13) "*Then, well, I searched the internet for 'learning disorders,' and boom, what comes up first? ADHD. I thought, what is this thing? And so I started to look a little at the explanations, the definitions of ADHD, and there were certain traits that reminded me of my child. And from there, I dug deeper, looked into who to consult, etc."*  (M9) "*But it's true that the diagnosis clarifies things. And it adds weight because, you know, people often think parents exaggerate... like, 'yeah, sure, my child is hyperactive,' but actually, yes, it’s true. So at least now we have a clinical assessment that proves it."*  (M2) *"But I would say... there isn’t much guidance. When you type ADHD into Google, they don’t tell you that you need to see a child psychiatrist to analyze it, that hospitalization might be needed, or that tests should be done—they don’t tell you any of that."*  (M10) *"I did some research on the internet, and the teacher had also guided me a bit by mentioning that for ADHD, assessments could be done privately, so I looked it up online."*  (F18) *“I let the mother manage, frankly the mother managed a lot, which she blamed me a little for not having participated enough”* |

1. **Internet, knowledge and the supporting role.**

| *A horizontal experience: to share and help each other.* | (M2) *“I immediately joined the ADHD France group on Facebook and now we understand better, now you understand better. (...) We are informed, helped, guided. On this group more than on Google. We are well guided, well informed: this is what to do, where to go”*  (M6) *“So now I'm going first to the group to already see and because on the sites I think we quickly got around to it anyway because well it's the same on each site”*  *(M11) "Then I go on forums, I listen, I read what other parents are saying, and I compare it to my son’s condition. So, the forums, it’s real-life experiences, you know it’s people who have been through it. I spent hours, really, hours reading all of that, and what helped me the most were the forums."*  (M13) “*I also read parents' opinions on the forums yeah, and there were also sites eh so but I preferred the forums to have an experience, the experiences of other families in fact”*  (M10) *"But it’s true that when moms share their daily experiences and give their little tips, like game ideas or using certain resources, you think, ‘Okay, I’ll try this kind of thing, and maybe it’ll work, or maybe not, we’ll see.’ So, these little tips make you think, ‘Well, she has a similar daily life to mine, so maybe it’ll work for us too.”*  (M8) "*So, she occasionally posts updates, and it’s true that I’ve been following her from the very beginning, and it’s nice to see that there is progress.”* |
| --- | --- |
| *Experience of relying on online resources for practical and official ADHD information* | (F17) *“We must learn not to generalize testimonies or behaviors and rather manage to find the official source”*  (F16) *“ADHD is really having the definition etc. to know what it is then, then for the drugs, it's also the role of the drug, why it is used, it's not the opinions to see if it works well. This is not the first research. It’s really: what is it?”*  (F17)*“There is no absolute truth, each case is different, each child is different, each family feeling is different, even if the cases are similar, the perception of those around them is also different and that can lead to characterizing a situation in a certain way when it is not”*  (F19) “*The first research you do is to understand the overall ADHD system, to know what it is exactly, what is happening in your child's brain (…) And then you are also confronted at certain times with testimonies which can increase your anxiety by saying to yourself we are never going to get out of this, but you have seen this thing it is even worse. But for me to read even worse things doesn't reassure me, it really worries me (…) For me the main danger of the Internet is people's judgments and the torrent of emotions and anguish when the sites are only receptacles of anguish, distress, and despair. I can’t do it, it’s too violent, it’s too difficult”.* |

1. **Internet and discordant discourse on ADHD between health professionals.**

| *Experiencing contradictory discourses* | (M7) *"So we are really alone in dealing with this, and so I looked into everything: gifted children, ADHD—I mean, I searched for everything. I was searching for myself because no one was giving me answers and no one could help her. So, overall, it’s been a long journey, honestly, it’s really tough."*  (M15) *“And here we are confronted with a double discourse. That is to say the discourse of neuropsychiatrists and the discourse of psychotherapists with a psychoanalytic tendency. And who therefore absolutely do not have the same vision of ADHD”*  (M9) *"But it’s true that there was a bit of wandering, you know... you feel like you’re left on your own, going from one professional to another without really knowing, without receiving any guidance."*  (M15) *"I re-registered for a while—I had left before because I needed to take some distance from all these issues that were becoming very overwhelming. And then, there was also the feeling of seeing children only through that lens, which wasn’t easy."* |
| --- | --- |
| *Experience of mismatched expectations and uncertainty* | (M9) *"We went through several psychologists because each time, it felt like we were going in circles; the advice (received from social network) wasn’t getting us anywhere."*  (M5) *"All this to say that after everything, I felt like it wasn’t leading anywhere, there wasn’t really a diagnosis for my son, so I put things on hold for a few months because I was just tired, really—I was trying to do what I could."* |
